# UNAGI: an automated pipeline for nanopore full-length cDNA sequencing uncovers novel transcripts and isoforms in yeast

**DOI:** 10.1007/s10142-020-00732-1

**Published:** 2020-01-18

**Authors:** Mohamad Al kadi, Nicolas Jung, Shingo Ito, Shoichiro Kameoka, Takashi Hishida, Daisuke Motooka, Shota Nakamura, Tetsuya Iida, Daisuke Okuzaki

**Affiliations:** 1 Department of Bacterial Infections, Research Institute for Microbial Diseases, Osaka University, Osaka, 565-0871 Japan; 2 Department of Infection Metagenomics, Research Institute for Microbial Diseases, Osaka University, Osaka, 565-0871 Japan; 3 Cykinso, Inc., Tokyo, 151-0053 Japan; 4 Department of Molecular Biology, Graduate School of Science, Gakushuin University, Tokyo, 171-0031 Japan; 5 Genome Information Research Center, Research Institute for Microbial Diseases, Osaka University, Osaka, 565-0871 Japan; 6 Integrated Frontier Research for Medical Science Division, Institute for Open and Transdisciplinary Research Initiatives, Osaka University, Osaka, 565-0871 Japan; 7 Single Cell Genomics, Human Immunology, WPI Immunology Frontier Research Center, Osaka University, Osaka, 565-0871 Japan; 8 Present Address: Genome Information Research Center, Research Institute for Microbial Diseases, Osaka University, Yamadaoka 3-1, Suita City, Osaka Japan

**Keywords:** Nanopore sequencing, Annotation, Isoforms, Differential gene expression, Stranding, Illumina, Full-length cDNA

## Abstract

Sequencing the entire RNA molecule leads to a better understanding of the transcriptome architecture. SMARTer (Switching Mechanism at 5′-End of RNA Template) is a technology aimed at generating full-length cDNA from low amounts of mRNA for sequencing by short-read sequencers such as those from Illumina. However, short read sequencing such as Illumina technology includes fragmentation that results in bias and information loss. Here, we built a pipeline, UNAGI or UNAnnotated Gene Identifier, to process long reads obtained with nanopore sequencing and compared this pipeline with the standard Illumina pipeline by studying the *Saccharomyces cerevisiae* transcriptome in full-length cDNA samples generated from two different biological samples: haploid and diploid cells. Additionally, we processed the long reads with another long read tool, FLAIR. Our strand-aware method revealed significant differential gene expression that was masked in Illumina data by antisense transcripts. Our pipeline, UNAGI, outperformed the Illumina pipeline and FLAIR in transcript reconstruction (sensitivity and specificity of 80% and 40% vs. 18% and 34% and 79% and 32%, respectively). Moreover, UNAGI discovered 3877 unannotated transcripts including 1282 intergenic transcripts while the Illumina pipeline discovered only 238 unannotated transcripts. For isoforms profiling, UNAGI also outperformed the Illumina pipeline and FLAIR in terms of sensitivity (91% vs. 82% and 63%, respectively). But the low accuracy of nanopore sequencing led to a closer gap in terms of specificity with Illumina pipeline (70% vs. 63%) and to a huge gap with FLAIR (70% vs 0.02%).

**Electronic supplementary material:**

The online version of this article (10.1007/s10142-020-00732-1) contains supplementary material, which is available to authorized users.

## Introduction

RNA sequencing (RNA-seq) is a revolutionary tool for transcript quantification, differential gene expression analysis, and transcript reconstruction and allows for the discovery of novel transcripts (Wang et al. 2009). Usually, the procedure requires converting mRNA to cDNA (Conesa et al. 2016) and stranded sequencing is possible using commercial kits like TruSeq (Sultan et al. 2012). SMARTer (Switching Mechanism at 5′-End of RNA Template) is a technology aimed at generating full-length cDNA from low amounts of mRNA for sequencing by short-read sequencers such as those from Illumina (Bostick et al. 2016). These sequencers require fragmented cDNA for sequencing (Conesa et al. 2016; Wang et al. 2009), which leads to loss of useful information, such as the strand of the reads. It also causes computational challenges in transcript reconstruction (Steijger et al. 2013). Sequencing bias, uneven read coverage, and uncovering splicing junctions are factors that lower the accuracy of transcriptome assembly (Oikonomopoulos et al. 2016). In particular, eukaryotic transcripts exhibit a high diversity that is difficult to uncover by traditional short-read sequencing methods (Pelechano et al. 2013). Additionally, losing strand information leads to less accurate transcriptome assembly and gene quantification (Zhao et al. 2015). Although SMARTer technology provides a stranded kit for depleted rRNA samples (Palomares et al. 2019) have shown that the stranded kit underperforms compared with that of the unstranded SMARTer kit. Recently however, new long-read sequencing technologies have been introduced such as Oxford Nanopore Technologies (ONT) that can sequence long reads up to 882 kb (Jain et al. 2018). MinION was the first sequencing device introduced by ONT (Ip et al. 2015) and was aimed at overcoming the aforementioned shortcomings of short-read sequencers.

Transcript reconstruction defines the precise boundaries of all transcripts and isoforms in an RNA sample (Garber et al. 2011). For short reads, the Tuxedo package, which is among the most popular tools, performs the reconstruction using Cufflinks or its successor StringTie. For long reads, transcriptome assembly is done either de novo without a reference or de novo with the guidance of a reference genome in case of its presence (Zhao et al. 2019). Tools for genome-guided transcriptome reconstruction using long reads are designed primarily for Pac Bio long reads and not ONT reads (Bayega et al. 2018). These tools rely simply on collapsing redundant isoforms (Abdel-Ghany et al. 2016; Wan et al. 2019) such as flair (Tang et al. 2018) without focusing on intronless genes. This can be challenging in reconstructing transcripts from dense genomes like the yeast’s and many lncRNAs may stay “hidden” under the shadow of neighbor protein-coding genes.

**Fig. 1:**
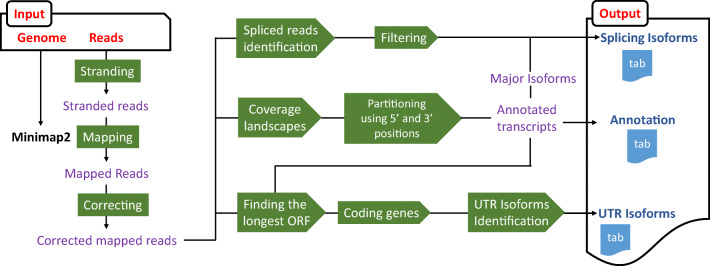
Schematic overview of the UNAGI pipeline. Reads from the ONT MinION are first stranded by looking for poly(A) or poly(T) tails at the ends and are separated into two files, sense and antisense. Those reads are then mapped to the genome using Minimap2 and their sequence is corrected using the genome. From these results, spikes and drops in coverage are identified as transcriptional unit boundaries as are spikes in number of 5′ or 3′ sites. The reads are also parsed looking for their splicing information and for long open reading frames (ORFs), allowing for the detection of isoforms. When several isoforms are discovered, only the major isoforms are annotated in the main output while all isoforms are listed in specific outputs

Herein, we developed a method that sequences full-length cDNA generated by SMARTer with nanopore sequencing and includes a pipeline to process generated reads and reconstruct the transcriptome. Using our method (Fig. 1), we sequenced RNA from two different biological samples of *Saccharomyces cerevisiae* (haploid and diploid cells) and evaluated this method in terms of gene quantification, differential gene expression, and transcript reconstruction. The evaluation was performed by comparing with another long read tool, FLAIR, and the data of Illumina sequencing of the same samples and a subsequent standard pipeline, StringTie.

## Results

### Sequencing

Reads from the ONT MinION were base-called and demultiplexed using albacore software. Overall, we obtained 11,022,685 reads comprised of 9.23 billion bases (Gb) for all four replicates (Additional file 1: Table S1 for details). The total N50 (the middle of the cumulative length) was 885 bases. High-quality reads were trimmed and aligned to the *S. cerevisiae* genome and transcriptome; 98.38% of the reads on average were aligned to the genome while only 88.91% were aligned to the transcriptome (Additional file 1: Table S2 for details). Reads were processed with our pipeline and the strand orientation was recovered for ~ 60% of the reads; these reads had similar alignment rates to the unstranded reads. Illumina sequencing with the HiSeq 2500 generated a total of 71,223,553 reads corresponding to 5.34 Gb for all four replicates (Additional file 1: Table S1 for details). These reads were aligned to the *S. cerevisiae* genome and transcriptome; 97.88% were aligned to the genome while only 72.98% were aligned to the transcriptome.

### Gene expression quantification

**Fig. 2:**
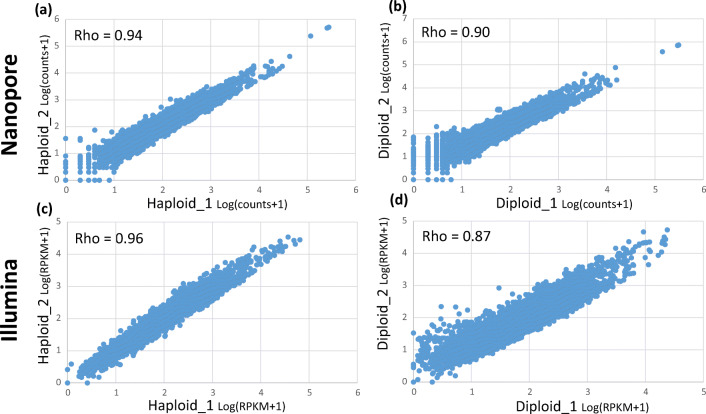
Correlation between biological samples. **a** Correlation of nanopore reads between the biological samples of haploid cells. **b** Correlation of nanopore reads between the biological samples of diploid cells. **c** Correlation of Illumina reads between the biological samples of haploid cells. **d** Correlation of Illumina reads between the biological samples of diploid cells. Rho indicates Spearman’s rank correlation coefficient. *X*- and *Y*-axes are the logarithmic (to base 10) transformation of counts + 1 for nanopore reads or RPKM (read per kilobase per million) for Illumina reads

Using the reads aligned to the *S. cerevisiae* transcriptome, we counted the aligned reads for each gene. As an indicator of quantification quality, we measured the correlation between biological samples. More correlation between biological samples indicates a higher accuracy in gene quantification. Spearman’s rank correlation coefficients of nanopore counts were 0.94 and 0.90 for the biological replicates of haploid and diploid cells, respectively (Fig. 2). Spearman’s rank correlation coefficients for reads per kilobase per million (RPKM) values of Illumina data were 0.96 and 0.87 for the biological samples of haploid and diploid cells, respectively (Fig. 2).

### Differential gene expression analysis

**Fig. 3:**
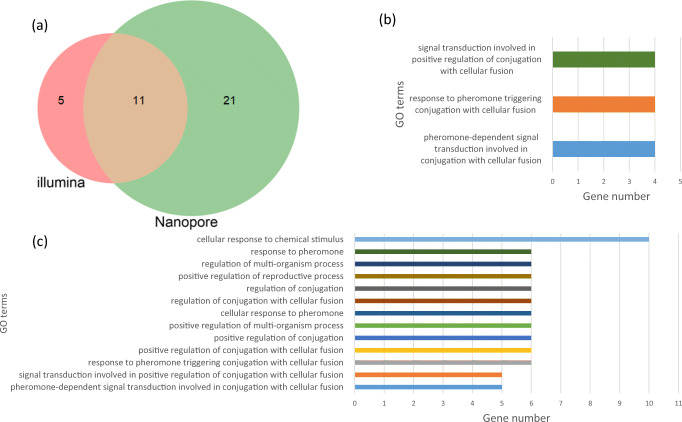
Differential gene expression between haploid and diploid cells. **a** Significantly DEGs by Illumina and nanopore sequencing. **b**, **c** Enriched GO terms of DEGs for Illumina (**b**) and nanopore (**d**) data

The main goal of gene quantification is to detect differentially expressed genes (DEGs) across biological samples. To perform differential gene expression analysis, we used the raw counts from both nanopore and Illumina data as input for the DESeq2 tool of the R/Bioconductor package. Stranded reads should have more information than do unstranded reads, so we performed the analysis with the nanopore standard reads. The Illumina data showed 16 significantly DEGs between haploid and diploid cells (Additional file 1: Table S3) while the nanopore data showed 32 significantly DEGs (Fig. 3 and additional file 1: Table S4). To investigate why Illumina failed to detect 21 out of 32 DEGs, we examined these genes individually. Twelve genes were found to have transcripts in the opposite direction. For example, *IME4* is a protein-coding gene that is only expressed in diploid cells; however, at the same locus, there is a gene (*RME2*) that codes for a noncoding RNA that is only expressed in haploid cells (Additional file 1: Figure S1). Illumina could not distinguish between the transcripts of these two different genes. Four other genes had homologous counterparts (*DDI2*, *DDI3*, *SOR1*, and *SOR2*) and both nanopore and Illumina alignments showed low-mapping quality. To confirm the reliability of differential gene expression analysis, we examined the mating and type-specific genes. These genes are differentially expressed between haploid and diploid cells. Thus, detecting differential expression for these genes by a specific method is an indicator of the reliability of this method. Using nanopore sequencing, we were able to detect all mating and type-specific genes but Illumina failed to detect 6 out of 11 genes (Table 1). All mating and type-specific genes that were detected as differentially expressed by nanopore but not by Illumina sequencing had transcripts on the opposite strand. To validate these results, we performed real-time PCR assays for genes that were found to be differentially expressed by Illumina, nanopore, or both. Differential expression was found for all genes except 1, 2, and 1 genes that were found to be differentially expressed by Illumina, nanopore, and both sequencers, respectively (Additional file 1: Figure S2). However, for all these genes, qPCR showed high expression for one of the two biological duplicates.

**Table 1:** Differential expression of mating and type-specific genes as detected by nanopore and Illumina sequencing. Genes were considered differentially expressed when the log2 fold change was > 2 with a *p* value < 0.05

Gene symbol	Description	Illumina	Nanopore
*STE5*	Pheromone-responsive MAPK scaffold protein	NDE*	DE**
*STE2*	Receptor for alpha-factor pheromone	DE	DE
*STE18*	Forms a dimer with Ste4p to activate the mating signaling pathway	DE	DE
*PRM2*	Pheromone-Regulated Membrane protein	NDE	DE
*MFA1*	Mating pheromone a	DE	DE
*MFA2*	Mating pheromone a	DE	DE
*RME2*	Regulator of Meiosis 2	NDE	DE
*IME4*	Inducer of meiosis	NDE	DE
*NEJ1*	Regulation of nonhomologous end joining	DE	DE
*HO*	Required for gene conversion at the MAT locus	NDE	DE
*FAR1*	CDK inhibitor and nuclear anchor	NDE	DE

*NDE indicates that the method found the gene not differentially expressed

**DE indicates that the method found the gene differentially expressed

### Transcript reconstruction assessment

**Fig. 4:**
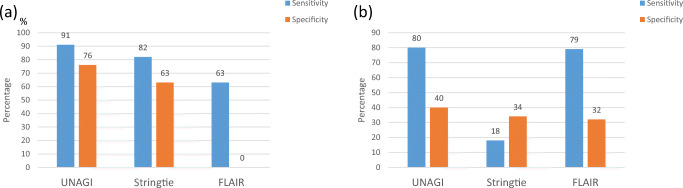
Transcriptome assessment for UNAGI, StrigTie and FLAIR. Sensitivity and specificity comparison isoform level (**a**) and transcript level (**b**)

We assembled the transcriptome with our pipeline UNAGI (https://github.com/iMetOsaka/UNAGI) using the coverage, the 3′ and 5′ genomic positions and the splice sites of nanopore reads. The provided coverage was more even and uniform than the coverage from Illumina reads (Additional file 1: Figure S3), which also lacked the 3′ and 5′ genomic positions of RNA transcripts. To assess our transcript reconstruction, we also reconstructed the transcriptome using a standard tool for Illumina data, StringTie, and a tool that is used with long reads, FLAIR. We compared the sensitivity and specificity across two levels, isoforms and transcripts. Assessment at the isoform level reflects the ability to assemble exons correctly. The number of annotated expressed spliced genes was 252 for the nanopore and 263 for Illumina. With UNAGI, the number of correctly assembled isoforms in the gene annotation was 229 out of 328 discovered spliced genes. Therefore, the sensitivity was 91% and the specificity was 70%. With FLAIR, only 159 isoforms were correctly assembled out of 5610 discovered spliced genes, corresponding to sensitivity and specificity of 63% and 0.02%. Upon close examination, we found that the reason for FLAIR low performance is due to nanopore reads’ low accuracy. It resulted in creating isoforms with false extra exons and false variants of the splicing sites. Using StringTie, the number of correctly assembled isoforms was 216 out of 342, corresponding to a sensitivity and specificity of 82% and 63%, respectively (Fig. 4a). At the transcript level, UNAGI detected 4367 annotated genes, FLAIR detected 4304, while StringTie detected only 989 annotated genes. The total number of discovered transcripts was 10,997, 13,397, and 3429 using UNAGI, FLAIR, and StringTie respectively. Accordingly, the sensitivity and specificity at the transcript level were 80% and 40% for UNAGI, 79% and 32% for FLAIR, and 18% and 34% for StringTie, respectively (Fig. 4b). We checked the duplicated transcripts, transcripts that represent the same gene with slight difference, and found 3394 and 744 duplicated transcripts in FLAIR and UNAGI, respectively (Additional file 1: Figure S4). Via UNAGI and FLAIR, 3877 and 6283 unannotated transcripts were discovered, respectively. While StringTie discovered only 175 unannotated transcripts. To evaluate unannotated transcript discovery with Illumina data more accurately, we ran StringTie with the guidance of an already known annotation; as a result, the number of discovered unannotated transcripts was 238.

### Assembled transcriptome

**Fig. 5:**
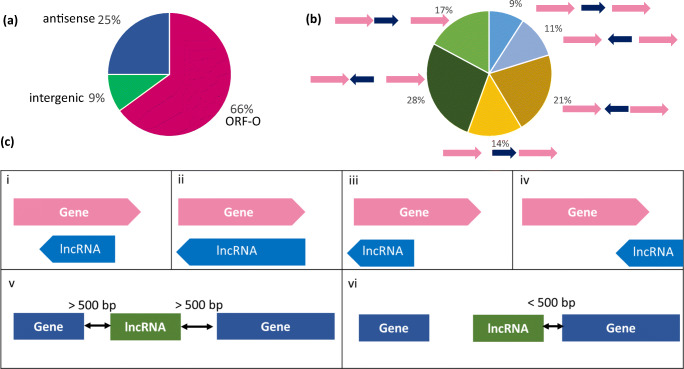
Type of novel transcripts. **a** Types of discovered transcripts: overlapping with ORF (ORF-O) of annotated genes, intergenic transcripts, and antisense transcripts. **b** Types of intergenic transcripts: intergenic transcripts (convergent, divergent), upstream transcripts (convergent, divergent), and downstream transcripts (convergent, divergent). **c** Types of novel transcripts: (i) antitranscript covering part of the ORF, (ii) antitranscript covering the whole ORF, (iii) antitranscript covering upstream the ORF, (iv) antitranscript covering downstream the ORF, (v) intergenic transcripts, and (vi) transcript covering the promotor of an annotated gene

UNAGI reconstructed 10,997 transcripts in total (Additional file 2); 63% of these transcripts overlapped with the open reading frame (ORF) of an annotated gene, either totally or partly and shared the same strand. Interestingly, many of these were overlapping with the 5′-UTR (Additional file1: Figure S5). The remaining transcripts (37%) were unannotated transcripts including 1282 intergenic transcripts (Fig. 5).

**Fig. 6:**
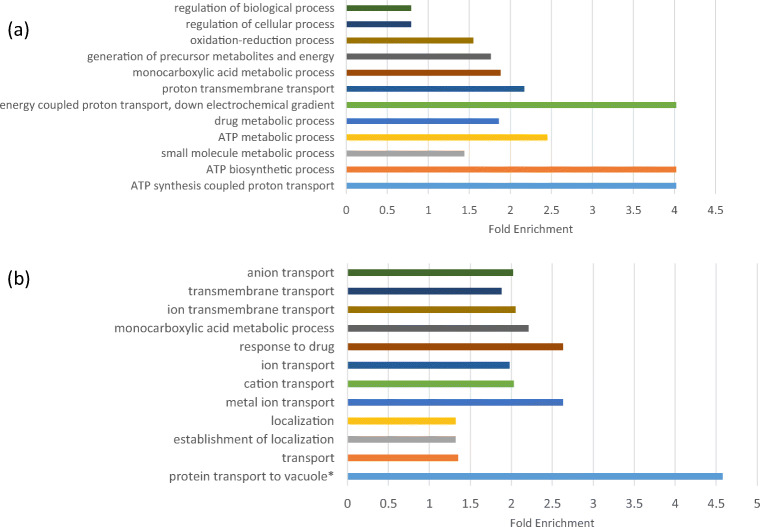
Enriched GO terms of sense genes of novel antitranscripts and closest genes of intergenic transcripts. **a** GO terms of genes that overlap with the novel noncoding transcripts, suggesting that these noncoding RNAs are mainly involved in the regulation of metabolism. **b** GO terms for the closest neighbor genes of the noncoding intergenic transcript, * protein transport to vacuole involved in ubiquitin-dependent protein catabolic process via the multivesicular body sorting pathway

To investigate the possibility of intergenic transcripts as protein-coding genes, we examined the longest ORF in transcripts and did not found any ORF with length more than 100 amino acids except 3 pseudogenes. To investigate the potential regulatory role of these transcripts, we analyzed the enriched gene ontology (GO) terms of the sense genes for our antisense transcripts and GO terms of the closest neighbor genes to our intergenic transcripts (Additional file 3). The majority of the enriched GO terms of sense genes were involved in metabolism especially ATP biosynthesis and energy (Fig. 6a). For intergenic transcripts, the majority were involved in transport especially in ions transport and protein transport that is involved in catabolism (Fig. 6b).

**Fig. 7:**
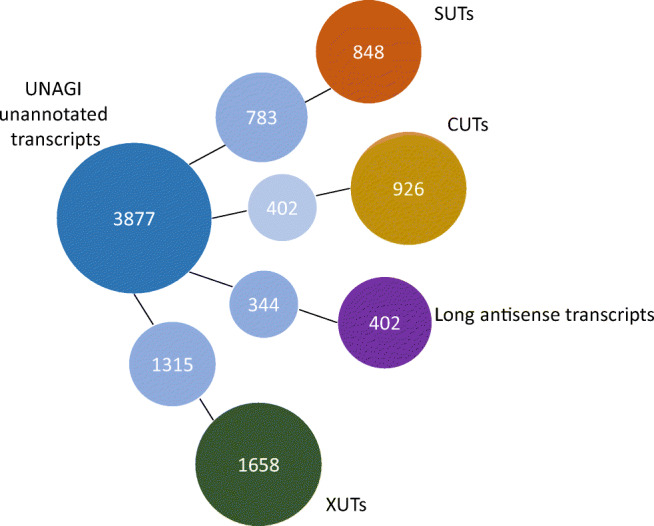
Novel transcript comparison with other resources. The number of UNAGI-unannotated transcripts on the left (dark blue circle), the number of common transcripts with other resources in the middle (light blue circles), and the number of other resource transcripts on the right (far right circles). SUTs, stable unannotated transcripts; CUTs, cryptic unstable transcripts; long unannotated antisense transcripts (Yassour et al. 2010); and XUTs, Xrn1-sensitive unstable transcripts

We compared unannotated transcripts with lncRNAs reported in other resources (van Dijk et al. 2011; Xu et al. 2009; Yassour et al. 2010) and found that 92% of stable unannotated transcripts (SUTs), 43% of cryptic unannotated transcripts (CUTs), 85% of antisense unannotated transcripts, and 79% of Xrn1-sensitive unannotated transcripts (XUTs) overlapped with our unannotated transcripts (Fig. 7). While 2182 transcripts did not overlap including 624 intergenic transcripts.

### Spliced genes

**Fig. 8:**
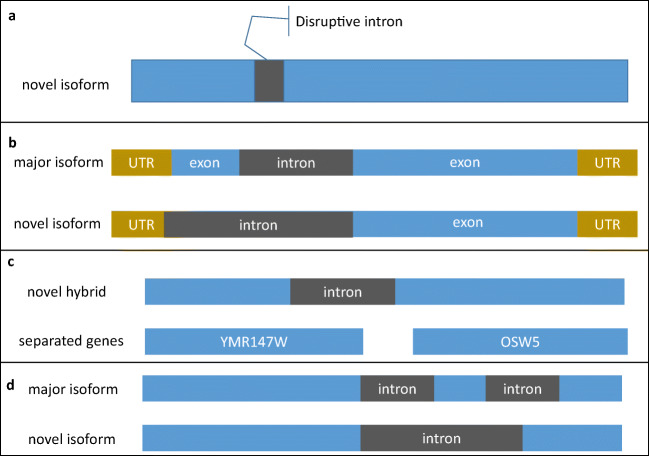
Types of novel splicing isoforms. **a** Intron disrupting the ORF(*HKR1* gene). **b** A novel isoform skips the first exon (*LSM2* gene). **c** Hybrid of two genes with splicing in the middle. **d** Novel isoform with an alternative site leading to skipping one exon

During the first step of alternative splicing profiling, UNAGI detected 625 splicing events, after which it filtered out 284 that had less than 1/10th of their locus coverage, leaving 341 splicing events. Some spliced genes had multiple alternative 5′ or 3′ sites but they were not considered and only 328 major isoforms were reported for UNAGI gene assembly (Additional file 4); 229 isoforms were annotated spliced genes and 112 were not. We analyzed these splicing events and found one annotated gene with a gene count less than the cut-off expression threshold, 23 annotated 5′ introns that were not in our reference databases, 45 novel splicing events in annotated genes, and 20 novel splicing events in intergenic transcripts. Some of the novel splicing events were disruptive for the ORF and occurred in the middle of the gene (Fig. 8a). Other isoforms skipped exons (Fig. 8b, d) and three isoforms included two genes, one of which included the ORFs of the two genes while the other two isoforms consisted of the promoter from the first gene and the ORF of the next gene (Fig. 8c).

We validated the discovered isoforms using the Illumina data. Most of the alternative isoforms were false positives resulting from insertion/deletion events. Three alternative isoforms (*NBL1*, *APE2*, and *LSM2)* however were supported by Illumina data. Only 10 novel splicing events in the coding genes were supported by Illumina data and the remaining were false positives; 28 of the false positives had low-mapping quality because they occurred in paralogous genes. The other false positives showed low levels of expression and for one gene, *HKR1*; one repeat was spliced out. Meanwhile, the Illumina reads were inconclusive because of the low-mapping quality of these tandem repeats. Similarly, only nine isoforms of long noncoding RNA (lncRNA) were supported by Illumina data.

Next, we investigated the false negatives, i.e., the undetected spliced genes, and found that 10 isoforms had reads with low-mapping quality, 9 of which were paralogous genes (9 isoforms of Y′ element ATP-dependent helicase), 5 genes were not spliced, 5 genes were fragmented and had low count, 1 gene was filtered out, 1 gene (*COX5B*) had a misidentified splicing site (Additional file 1: Figure S6), and 1 gene (*GCR1*) had a novel splicing site.

### 5′ and 3′-untranslated region (UTR) isoforms

We investigated the variations in the 5′-UTRs and considered the variants as 5′-UTR isoforms. We found 1089 genes with alternative 5′-UTR isoforms (Additional file 5) and the gene with the highest number of 5′-UTR isoforms was *RPL41B* (codes for a ribosomal protein) with 59 isoforms. However, the majority (90%) had less than 10 isoforms (Additional file 1: Figure S7). On average, each gene had 6 isoforms. One implication of these isoforms is upstream ORFs (uORFs) that exist within the 5′-UTR and may interfere with the main ORF translation. Therefore, we looked for uORFs in all annotated genes and found 1892 genes (34%) with uORFs within their 5′-UTRs, 169 of which showed alternative 5′ isoforms (Additional file 5). For example, *SNF11* had two 5′-UTR isoforms in our data, one with an uORF and the other without (Additional file 1: Figure S7).

We also analyzed the variations in the 3′-UTRs and considered the variants as 3′-UTR isoforms. We found 1482 genes with more than one alternative polyadenylation poly(A) site. The average number of alternative poly(A) sites was nine alternative sites per gene (Additional file 6).

### Benchmarking of UNAGI and FLAIR for mice long reads

Yeast genome is dense and the majority of genes are monoexonic. However, higher eukaryotes are in the contrary less dense and the majority of genes are composed of many exons. Therefore, we did benchmarking using mice long reads from brain cells (Sessegolo et al. 2019). Total reads were 1,267,830 high-quality corrected reads. FLAIR assembled 17,962 transcripts while UNAGI assembled 26,706 transcripts including 9104 transcripts supported by less than 3 reads. We assessed the reconstructed transcriptomes at two levels; junction and transcript levels. FLAIR identified 200,596 junction sites while UNAGI identified more than the double, 461,292 sites. Most of the sites that were not discovered by FLAIR were covered by few reads (~ 70% were covered by one read and ~ 18% were covered by two reads). At the transcript level, UNAGI assembled 21,684 transcripts compared with 20,717 transcripts assembled by FLAIR. Again, most of the genes that were not discovered by FLAIR had very low count.

## Discussion

We have shown a method for massively sequencing full-length cDNA generated by SMARTer technology using the long-read ONT nanopore sequencer MinION and built a pipeline to process the yielded reads, perform differential gene expression analysis, and reconstruct the transcriptome of studied samples. To evaluate the results, we also sequenced the full-length cDNA generated by SMARTer technology via Illumina sequencing and processed the data using a standard pipeline (the Tuxedo pipeline). The model we employed was the budding yeast *S. cerevisiae*, a well-studied eukaryotic model organism with extensive gene annotation (Jenjaroenpun et al. 2018).

A major setback of nanopore sequencing is its low accuracy compared with that of short-read sequencers like Illumina (Rang et al. 2018); however, this setback did not affect mapping reads using our method. We achieved similar and even slightly higher genomic mapping rates than that of the Illumina sequencer. We also found a difference between the mapping rates of the genome and transcriptome, suggesting that an important portion of the reads aligns to the intergenic region.

Both nanopore and Illumina sequencing methods captured the correlation between biological samples and achieved the same conclusion that haploid samples are more correlated than diploid samples. This suggests that quantification of nanopore reads is as good as that seen for Illumina sequencing and agrees with other studies that reported accurate gene expression quantification with nanopore cDNA sequencing (Byrne et al. 2017; Oikonomopoulos et al. 2016). Mating genes, cell type genes, and genes involved in pheromone response were the main DEGs between haploid and diploid cells (de Godoy et al. 2008; Haber et al. 2012; Li et al. 2010). Our method detected differential expression for all these genes, whereas Illumina sequencing failed to detect differential gene expression for some genes. Many studies reported that antisense transcription regulates mating and cell type-specific genes (Gelfand et al. 2011; Hongay et al. 2006; Lardenois et al. 2011; Moretto et al. 2018), which is consistent with our data showing many antisense transcripts at the locus of mating and type-specific genes; this masked differential gene expression when using Illumina’s strand-unaware gene quantification. Additionally, antisense reads came from neighbor genes that were differentially expressed. It has been shown before that stranded RNA-seq provides more accurate gene quantification than does unstranded RNA-seq (Zhao et al. 2015); similarly, our study highlighted the importance of stranded quantification for achieving accurate gene quantification and differential gene expression analysis.

Our pipeline UNAGI outperformed Illumina pipeline in transcriptome reconstruction across both isoform, and transcript levels and provided higher resolution in transcript reconstruction than could the Illumina pipeline annotations, StringTie. Moreover, the UNAGI-reconstructed transcriptome predicted most of the annotated transcripts correctly (80%) unlike StringTie, which only predicted 18% of the annotated transcripts. UNAGI was also superior in terms of specificity, providing a higher percentage of correctly predicted transcripts in the annotation (40% vs. 34%). The annotating process is more difficult with short reads because of the lack of full-length information. On the other hand, long reads retain the full-length of the transcripts, producing a less biased coverage (Additional file 1: Figure S2) and providing the 5′ and 3′-end positions for each transcript. This avoids the bias effect and resolves overlapping transcripts making the annotation process clearer. Although the last factor is more important in compact genomes like those of yeast, the human transcriptome showed similar sensitive and specificity with StringTie (Pertea et al. 2015). FLAIR as expected gave better results than Illumina. Still, UNAGI discovered more annotated transcripts and had better specificity than FLAIR. UNAGI is optimized for transcript reconstruction with a separate module for isoforms profiling unlike FLAIR and similar tools that are designed mainly for isoforms definition (Tang et al. 2018) which results in high number of reconstructed isoforms and reduced specificity.

Nanopore sequencing is a powerful method for isoform reconstruction, especially in the case of complex structures (Bolisetty et al. 2015). However, transcript structure is simple in *S. cerevisiae*, which is mostly comprised of two exons and an intron, and the portion of annotated spliced gene number is low. Still, our pipeline showed high sensitivity and outperformed StringTie. Specificity, on the other hand, was considerably lower than sensitivity but still higher than that of the Illumina tools. Surprisingly, UNAGI outperformed FLAIR at the isoform level. One weakness of nanopore sequencing is its low accuracy that leads to false negatives including false alternative isoforms and splicing events. FLAIR and other long read tools require reads correction to compensate for nanopore low accuracy (Zhao et al. 2019). But UNAGI filter out these false events based on long reads themselves.

Our pipeline discovered 19 novel isoforms including a 5′ prime intron (*SIP18*), a splicing event that joined two genes together (*OSW5* and *YMR147W*), a splicing event that joined one gene with the promoter of the upstream gene, and normal isoforms with two exons and one intron, which were the most frequent (Fig. 7). The *HKR1* protein product has 12 tandem repeats that cannot be detected in case of splicing by short reads, highlighting the power of long-read sequencing. Some of the discovered isoforms have been described before; for example, a recent study (Eisenberg-Bord et al. 2018) reported on the splicing event that connects *OSW5* and *YMR147W*. Most of the remaining isoforms were disruptive to the ORF, which has been suggested to result from stress conditions and as a mechanism to downregulate gene expression (Kawashima et al. 2014). In addition, some splicing events had multiple isoforms due to 5′ or 3′ alternative splicing sites. These isoforms were less frequent than the major isoform, possibly making them hard to detect via short-read sequencing. These findings are in agreement with other studies that also confirmed the occurrence of alternative isoforms in yeast (Kawashima et al. 2014; Schreiber et al. 2015). The 5′ and 3′-UTR isoforms also revealed the complexity of the transcriptome. Each gene had on average seven isoforms, considering both the 5′ and 3′-ends, and a similar diversity has been reported before (Pelechano et al. 2013). This finding has implications for translational regulation with different translation rates between different isoforms depending on the presence of uORFs, as in the case of the *SNF11* gene (Additional file 1: Figure S7).

Many of the predicted transcripts from UNAGI did not correspond to the annotated transcripts. One possible explanation is the pervasive nature of *S. cerevisiae* transcription (Xu et al. 2009), which was demonstrated in our results by the higher genomic mapping rate (> 90%) compared with the transcriptome mapping rate. Our automated pipeline discovered 3877 unannotated transcripts, including 1282 intergenic transcripts. We did not find long ORFs (> 100 amino acids) in these transcripts, suggesting that they are lncRNAs, if they have any functional roles. lncRNAs are transcribed by Polymerase II (Tuck and Tollervey 2013) and can thus be captured by our poly(A) selection-based protocol. However, their discovery by short-read annotating pipelines is limited because lncRNAs have low expression levels and lack translational features (Lagarde et al. 2017; Steijger et al. 2013). This was evident in our Illumina data as it discovered only 238 novel transcripts, even when it was guided by gene annotation. On the other hand, our pipeline UNAGI discovered five times that number of unannotated lncRNA transcripts, highlighting its high performance compared with that of Illumina methods in noncoding RNA discovery. Until recently, lncRNAs were thought to be random noise from the transcription process (Jensen et al. 2013). However, many of these RNAs have been reported to be involved in cell fate regulation (Gelfand et al. 2011; Moretto et al. 2018) and response to environmental change (Castelnuovo et al. 2013; Haber et al. 2012; Martens et al. 2004; Nevers et al. 2018; Yao et al. 2010), whether they are antisense or intergenic transcripts. Our analysis of the candidate regulated sense genes revealed they were mainly involved in metabolism and biosynthesis regulation. We also analyzed the closest neighbor genes of intergenic transcripts, if there were any, under the assumption they could be candidates of gene regulation. Our analysis revealed a high enrichment of the ion transmembrane transport and gene expression genes. This suggests that at least some of our novel transcripts have a regulatory role in the expression of these genes, since the type of candidates requires regulation in response to environmental change. Moreover, many of the unannotated noncoding RNAs, SUTs, CUTs (Xu et al. 2009), XUTs (van Dijk et al. 2011), and antisense transcripts (Bostick et al. 2016) overlapped with our unannotated transcripts, showing our method ability to discover lncRNAs compared with Illumina.

Finally, we benchmarked UNAGI using corrected long reads from mice (Sessegolo et al. 2019). Again, UNAGI showed higher performance using lower count threshold although it produced high number of transcripts compared with FLAIR. The threshold parameter can be adjusted depending on the coverage and intended purpose resulting in a greater flexibility for the reconstruction process.

In conclusion, our method recovered the strand orientation of long reads and proved comparably accurate with conventional sequencing methods using Illumina-generated short reads in terms of gene quantification and differential gene expression. UNAGI outperformed short read–based pipeline in transcript reconstruction due to higher sensitivity and specificity, discovered a large number of novel transcripts with a potential regulatory role, and revealed a complex transcriptional landscape. Moreover, comparing with another long read tool, it showed higher specificity at transcript and isoforms levels. However, the low accuracy of nanopore sequencing still poses a challenge, especially for isoform profiling.

## Methods

### Cell culture and RNA extraction

*Saccharomyces cerevisiae* BY4741 laboratory strain was kindly provided by Prof. Takashi Hishida (Haruta et al. 2012). The strains used in this study have the genotype *MATa his3Δ leu2Δ met15Δ ura3Δ rad14Δ::Kan* and *MATa/alpha his3Δ/his3Δ leu2Δ/leu2Δ met15Δ/met15Δ ura3Δ/ ura3Δ rad14Δ::Kan/rad14Δ::LEU2*. Both were cultured in 10 mL YDP (yeast extract peptone dextrose) medium overnight at 30 °C. Growth was stopped when the culture reached a density of 2 × 10^8^ cells/mL, after which RNA was extracted using the miRNeasy Mini Kit (Qiagen, Hilden, Germany) according to manufacturer’s instructions. Briefly, yeast cells were mechanically disrupted using glass beads and then ethanol was added to promote the binding of total RNA to the membrane. After binding, the RNA was washed and eluted with RNAse-free water. RNA quality and quantity were estimated using the NanoDrop Spectrophotometer (Life Technologies, Carlsbad, CA) and Agilent Bioanalyzer 2100 (Agilent Technologies, Santa Clara, CA).

### Library preparation and RNA-Seq using Illumina HiSeq2500

Starting with 10 ng of total RNA from two biological samples (haploid and diploid cells; four biological samples overall, two replicates per sample), we generated full-length cDNA using the SMARTer Ultra Low RNA Kit for Illumina Sequencing (Takara Bio, Kusatsu, Japan) according to manufacturer’s instructions. Briefly, samples were diluted with reaction buffer and primed with 3′ SMART-Seq CDS Primer II A. First-strand cDNA synthesis and cDNA amplification were performed in a single step as the master mix contained both SMARTScribe™ Reverse Transcriptase and SeqAmp DNA Polymerase with SMART-Seq HT Oligonucleotide. First-strand cDNA was synthesized at 42 °C for 90 min, after which the cDNA was amplified (initial denaturation at 95 °C for 1 min, followed by 8 cycles of denaturation at 98 °C for 10 s, annealing at 65 °C for 30 s, and elongation at 68 °C for 3 min, with a final elongation step at 72 °C for 10 min). The generated cDNA was purified using the Agencourt AMPure XP Kit (Beckman Coulter, Brea, CA), cDNA concentration was measured using the Qubit™ dsDNA HS Assay Kit (Life Technologies), and fragment sizes were analyzed with the LabChip ® GX Touch ™ nucleic acid analyzer (PerkinElmer, Waltham, MA). An Illumina library was prepared using the Nextera DNA Library Preparation Kit (Illumina, San Diego, CA) according to the SMARTer kit instructions. The cDNA was fragmented by tagmentation at 55 °C and then DNA was amplified (initial denaturation at 95 °C for 30 s, followed by 12 cycles of 95 °C for 10 s, 55 °C for 30 s, and 72 °C for 1 min, with a final elongation step at 72 °C for 5 min) to introduce sequencing adapters. Finally, cDNA was purified using Agencourt AMPure XP Kit (Beckman Coulter) and sequenced with HiSeq 2500 × 75 bp (Illumina).

### Library preparation and RNA-Seq using ONT MinION

Starting with 10 ng of the same RNA samples that had been used above, we generated full-length cDNA using SMARTer cDNA synthesis kits (Takara Bio) according to manufacturer’s instructions. To avoid chemical blocking of primers used in amplification, which would affect downstream nanopore library preparation, we did not use the full-length cDNA generated by the SMARTer Ultra Low RNA Kit. Briefly, RNA samples were primed with 3′ SMART-Seq CDS primer IIA and first-strand cDNA was synthesized using a SMARTScribe™ Reverse Transcriptase master mix. Then, cDNA was amplified using PCR primer II A and LongAmp® Taq DNA Polymerase (New England Biolabs, Ipswich, MA), after which it was purified with the Agencourt AMPure XP Kit. cDNA concentration was measured with the Qubit™ dsDNA HS Assay Kit and fragment sizes were analyzed using the LabChip analyzer. The sequencing library was prepared using Ligation Sequencing Kit SQK-LSK108 (Oxford Nanopore Technologies, Oxford, UK) with the native barcoding protocol according to manufacturer’s instructions. Briefly, the cDNA was end-prepped using the Ultra II End Prep Mix and then barcodes (1, 2, 3, and 6) from the Native Barcoding Expansion 1–12 were ligated using a Blunt/TA ligase master mix (New England Biolabs). Samples were pooled to a final volume of 51 μL. Finally, the sequencing adapters were ligated by Quick T4 DNA ligase (New England Biolabs) and the library was loaded onto a MinION flow cell (FLO-MIN106, R9.4). Sequencing was performed over 48 h. Reads were base-called with Albacore (version 2.3.1) (https://github.com/Albacore/albacore), after which low-quality reads were excluded and high-quality reads chosen. Nanopore adaptors were trimmed using Porechop with basic adapter trimming parameters.

### Real-time quantitative PCR

Total RNAs were treated with DNase I (Thermo Fisher Scientific K.K., MA, USA, 18068015). cDNA was synthesized with the ReverTra Ace Moloney murine leukemia virus reverse transcriptase with point mutations (TOYOBO CO., LTD., Osaka, JPN, FSQ-101) according to the manufacturer’s protocols. Quantitative RT-PCR was analyzed with TB Green Premix Ex Taq II Tli RNaseH Plus (TAKARA, Shiga, JPN, v201903Da) and LightCycler480 System II (Roche Diagnostics K.K., Rotkreuz, CHE) under the following condition: 95 °C 30 s − (95 °C 5 s − 60 °C 30 s) × 45 cycles. Detailed information about the primers used here is shown in Additional file 8. Data were normalized to the expression of ACT1 for each sample.

## UNAGI

To process the nanopore reads and assemble the transcriptome, we built a pipeline called UNAGI, which is short for UNAnnotated Gene Identifier and inspired by the Japanese word for freshwater eel. It was chosen because the mRNA is kept long, like an eel, without any fragmentation where the head and tail are distinguishable, unlike the standard procedure with a short-read sequencer. This method allows us to strand the reads and provides more useful information for the annotation process.

First, the pipeline uses fastq files as input and starts by stranding the reads. It then annotates the transcripts using a genome-guided de novo approach and proceeds to run isoform profiling (Fig. 1). The pipeline is written in python and executes shell commands for mapping with Minimap2, processing the alignment file with Samtools (Li et al. 2009) and Bedtools (Quinlan and Hall 2010).

### Stranding

The orientation of the RNA molecule is lost after generating and amplifying full-length cDNA as cDNA molecules contain both sense and antisense strands. Taking advantage of the ability of the ONT nanopore sequencer to sequence full-length cDNA, the orientation information can be retrieved by simply looking for the poly(A) tail and SMART oligonucleotides flanking the read. The pipeline analyses the ends of each read and sorts it to one file with its original sequence if there is a poly(A) tail at the 3′-end or to another file with its reverse-complemented sequence if it has poly(T) at the 5′-end. The read will be discarded in case of failing to detect either poly(A) or poly(T) at its ends. Tail sequences can be modified in the configuration file when using custom primers other than those of the SMARTer kit.

### Transcript reconstruction

The next step in our pipeline is aligning the stranded reads to the genome using Minimap2 (Li 2018) with long-read parameters (− *ax* splice − secondary = no). Then, the pipeline calculates genomic coverage per base and identifies the 5′ and 3′ genomic positions using Bedtools genomecov for positive and negative strands separately. This separation is crucial to prevent antisense transcript interference with annotating their counterpart sense transcripts. Finally, the pipeline annotates the genes using our algorithm. The algorithm uses the coverage first to produce transcriptional units; it scans the genomic coverage for each chromosome and records the start of a unit when coverage exceeds a pre-set threshold and records its end when coverage falls below the same threshold, storing the reads in this unit. These units may include more than one gene; for example, some genes may overlap, especially in dense genomes like those of yeast or in the case of bidirectional promoters. The 5′ and 3′ positions can help refine the produced units and separate any overlapping. The 3′-ends of one gene are not at the same exact position but dispersed over a small region and there is a need to cluster them to determine the transcript end site. The pipeline clusters 3′ positions that are close to each other (according to a pre-set threshold), after which it can separate the overlapped genes in an annotated unit by searching for 3′ cluster positions in the body of this unit as an indicator of the end of one gene and for 5′ positions for these reads to determine the beginning of the next gene in the unit.

For higher eukaryotes, where splicing events are pervasive, spliced reads are processed separately in the transcriptional units. Spliced genes are identified using their splicing sites by collapsing. Then transcripts with similar and or identical splicing sites are clustered together.

### Isoform profiling

Protein-coding genes possess many different isoforms. The pipeline searches for the longest ORF in each annotated gene and considers the ones that are more than 100 amino acids in length. Alternatively, gene annotation can be provided to the pipeline. In both cases, the pipeline looks for the following types of isoforms:

#### 1. Alternative splicing isoforms

Nanopore long-read alignments represent the full transcript and span all its comprising exons and introns; thus, they can be considered representative of the actual splicing isoforms without needing any assembly, contrary to short reads. The pipeline simply clusters reads with identical splicing sites. Starting from the alignment file as input and using Bedtools bamToBed (with -split option), the alignment file format (bam) is converted to the more concise bed format, which can report each portion of the split alignment as a distinct interval. Using this property, the pipeline can extract split alignments and cluster those with identical split sites together. However, the low accuracy of nanopore reads may result in false negatives. Because of the random occurrence of this kind of error, the same error tends not to occur in multiple reads. Accordingly, the pipeline considers isoforms supported by more than one read, followed by secondary filtering for isoforms that are supported by reads less than 1/10th the coverage of the genomic locus. Another problem to account for is the poly(A) tails used for stranding the reads. They are sometimes mistakenly mapped to separate regions as new exons. To address this problem, the pipeline searches for short exons with poly(A) sequences and removes them from the pool. For gene annotation, in the case of multiple alternative isoforms for the same splicing event, the pipeline defines the exon boundaries at the highest frequency splice site and retains the other isoforms in a separate file. The main result file reports only major isoforms and presents them as combinations of exons with gene annotations.

#### 2. 5′-UTR isoforms

The pipeline searches in the same strand as the gene for reads that start at or after the gene start site and span the first codon of the main ORF. In the case of providing the annotations without 5′-UTRs, it takes the reads that span the start codon (the read starts before the first codon and ends after the first codon). The 5′ positions of these reads are considered alternative start sites if their coverage exceeds a pre-set threshold, and in the case of lacking gene start information, the farthest 5′-end with coverage more than the pre-set threshold is considered the next gene start.

#### 3. 3′-UTR isoforms

Similarly, the pipeline searches for reads of the same strand as the gene that ends before the gene end site and spans the last codon of the main ORF. In the case of lacking 3′-UTR information, the pipeline calculates 3′-UTR as before. The 3′ positions of these reads are considered alternative end sites if their coverage exceeds a pre-set threshold.

### Quantification and differential gene expression analysis

Raw reads were aligned against the *S. cerevisiae S288C* transcriptome (assembly R64) using Minimap2 with parameters set for Illumina short reads or nanopore long reads, after which the aligned reads for each gene were counted. In the case of stranded data, we excluded reads with opposite orientation and only considered aligned reads with the same strand as the counted genes. For quantification of Illumina data, we calculated RPKM. We used the R/Bioconductor packages of the DESeq2 tool (Anders and Huber 2010) to model the counts of nanopore and Illumina data following negative binomial distribution and analyze differential gene expression between the two biological samples. We considered genes with a log2 fold change > 2 (*p* < 0.05 according to the Wald test performed by DESeq2) as significantly differentially expressed.

### Transcript reconstruction

Nanopore reads from the diploid sample were processed using our pipeline UNAGI. To assess our transcript reconstruction, the transcriptome was reconstructed using Illumina reads with StringTie without providing gene annotation. Tophat2 was used to align the reads and used the alignment as input for StringTie with the default parameters. Stranded long reads were also processed using another long read tool, FLAIR (Tang et al. 2018), using the recommended parameters. Correction step was skipped.

### Transcript reconstruction assessment

We calculated sensitivity and specificity for our assembly and other pipeline assemblies at the isoform (intron) and transcript levels. The reference gene annotation included genes that were expressed in more than four transcripts by nanopore sequencing (5476 genes) and in more than four RPKM by Illumina sequencing (5894 genes). These genes were used as a reference in the pipeline performance assessments, each with its corresponding expressed genes. For the isoform level, we assessed the ability of each pipeline to assemble the annotated introns correctly. Sensitivity was calculated as the number of genes with correct intronic structure divided by the number of actual genes with intronic structure. Specificity was calculated as the number of genes with a correct intronic structure divided by the number of genes with a predicted intronic structure. For the transcript level, we set loose criteria for the start and end sites of transcripts as we did not have a reliable database regarding UTRs. A transcript was considered correct if it overlapped with an annotated gene at up to 40% difference from each end. We used this approach instead of overlapping with the ORF to avoid false positives with artifact fusion transcripts. Sensitivity was calculated as the number of correct transcripts divided by the number of actual expressed genes and specificity was the number of correct transcripts divided by the number of predicted transcripts. For mice data, we count the correct junction sites.

### Unannotated transcripts

Transcripts were compared with the Saccharomyces Genome Database (SGD) annotation (Additional file 7) to find the unannotated transcripts. These transcripts were considered to assess the ability of each pipeline to find novel transcripts. In addition, the unannotated transcripts were compared with other reports. GO terms analysis was done using PANTHER tool with Fisher’s exact test and we considered only results with false discovery rates less than 0.5 (Mi et al. 2019).

## Electronic supplementary material

ESM 1 (DOCX 724 kb).

ESM 2 (CSV 649 kb).

ESM 3 (CSV 6 kb).

ESM 4 (XLSX 27 kb).

ESM 5 (XLSX 272 kb).

ESM 6 (CSV 360 kb).

ESM 7 (TXT 4044 kb).

ESM 8 (XLSX 14 kb).

## Data Availability

The datasets that support the conclusions of this article are available in the DDBJ database (BioProject PRJDB8048) under the accession codes DRR170478-DRR170485. UNAGI was developed using Python language. The source code is available at the GiHub repository (https://github.com/iMetOsaka/UNAGI).

## Abbreviations

cDNA: Complementary DNA
GO: Gene ontology
kb: Kilobase
mRNA: Messenger RNA
PCR: Polymerase chain reaction
bp: Base pair
CUTs: Cryptic unannotated transcripts
DEG: Differentially expressed genes
lncRNA: Long noncoding RNA
ONT: Oxford Nanopore Technology
ORF: Open reading frame
RPKM: Reads per kilobase per million
SUTs: Staple unannotated transcripts
UNAGI: UNAnnotated Gene Identifier
uORF: Upstream open reading frame
UTR: Untranslated region
XUTs: Xrn1-sensitive unstable transcripts
