# Supplementary material for: UNAGI: an automated pipeline for nanopore full-length cDNA sequencing uncovers novel transcripts and isoforms in yeast
Source: Funct Integr Genomics. 2020 Jan 18;20(4):523–36. doi: 10.1007/s10142-020-00732-1 (PMC7283198; doi:10.1007/s10142-020-00732-1)

**Supplementary Table 1.** Sequencing yield from Nanopore and Illumina sequencing

| **Platform** | **Haploid cells -rep1** | **Haploid cells -rep2** | **Diploid cells -rep1** | **Diploid cells -rep2** |
| --- | --- | --- | --- | --- |
| **Nanopore** | 1.68 Gb* | 1.93 Gb | 1.32 Gb | 2.86 Gb |
|  | 2,221,335 reads | 2,558,331 reads | 1,545,574 reads | 3,970,029 reads |
| **Illumina** | 1.36 Gb | 1.21 Gb | 1.34 Gb | 1.44 Gb |
|  | 18098789 reads | 16171835 reads | 17809261 reads | 19143668 reads |
| ***** Giga base pairs | | | | |

**Supplementary** **Table 2.** Mapping rate of Nanopore and Illumina reads to the *Saccharomyces cerevisiae* genome and transcriptome (assembly R64)

| **Platform** | **Reference** | **Average** | **Haploid cells -rep1** | **Haploid cells -rep2** | **Diploid cells -rep1** | **Diploid cells -rep2** |
| --- | --- | --- | --- | --- | --- | --- |
| **Nanopore** | **Transcriptome** | 88.91 % | 85.92 % | 91.31 % | 90.52 | 87.89 % |
|  | **Genome** | 98.38 % | 98.15 % | 98.59 % | 98.53 % | 98.25 % |
| **Illumina** | **Transcriptome** | 72.98 % | 68.65 % | 74.23 % | 79.43 % | 69.62 % |
|  | **Genome** | 97.87 % | 98.18 % | 98.15 % | 97.33 % | 97.85 % |

Supplementary table 3. Significantly differentially expressed genes as detected by Illumina

| **Gene Symbol** | **Description** | **Log2 Fold change** | **p-value** |
| --- | --- | --- | --- |
| *LEU2* | 3-isopropylmalate dehydrogenase | 12.02808 | 0.000000 |
| *FDH1* | formate dehydrogenase (NAD+) | 4.762093 | 0.000000 |
| *HPF1* | mannoprotein | 2.336745 | 0.000021 |
| *PDR12* | ATP-binding cassette multidrug transporter PDR12 | 2.077566 | 0.000025 |
| *MHF2* | Mhf2p | -2.05015 | 0.000573 |
| *YNL146W* | hypothetical protein | -2.29276 | 0.000383 |
| *STE2* | alpha-factor pheromone receptor STE2 | -2.32761 | 0.000003 |
| *YLR227W-B* | gag-pol fusion protein | -2.33644 | 0.000000 |
| *GAT4* | Gat4p | -2.37104 | 0.005095 |
| *MFA1* | mating pheromone a | -2.39792 | 0.000014 |
| *YNL146C-A* | hypothetical protein | -3.20891 | 0.000000 |
| *ICS2* | Ics2p | -3.5336 | 0.000000 |
| *STE18* | Ste18p | -3.70121 | 0.000000 |
| *RDT1* | Rdt1p | -3.84995 | 0.000000 |
| *NEJ1* | Nej1p | -4.34221 | 0.000000 |
| *MFA2* | mating pheromone a | -7.06367 | 0.000000 |

Supplementary table 4. Significantly differentially expressed genes as detected by Nanopore

| **Gene Symbol** | **Description** | **Log2 Fold change** | **p-value** |
| --- | --- | --- | --- |
| *LEU2* | 3-isopropylmalate dehydrogenase | 7.452241 | 0.00000 |
| *IME4* | mRNA (N6-adenosine)-methyltransferase | 6.081181 | 0.00009 |
| *FDH1* | formate dehydrogenase (NAD+) | 5.043203 | 0.00000 |
| *HPF1* | mannoprotein | 4.410114 | 0.00000 |
| *DDI3* | cyanamide hydratase | 2.608226 | 0.00740 |
| *RCN1* | Rcn1p | 2.600942 | 0.00232 |
| *DDI2* | cyanamide hydratase | 2.582143 | 0.00816 |
| *YPL277C* | hypothetical protein | 2.179766 | 0.00286 |
| *UTP18* | Utp18p | 2.113104 | 0.03817 |
| *YKL068W-A* | hypothetical protein | -2.06857 | 0.00445 |
| *SCR1* | SCR1 | -2.09563 | 0.00169 |
| *MFA1* | mating pheromone a | -2.17855 | 0.00253 |
| *STE5* | Ste5p | -2.30126 | 0.00076 |
| *HAP1* | Hap1p | -2.41115 | 0.00000 |
| *STE2* | alpha-factor pheromone receptor STE2 | -2.44574 | 0.00005 |
| *GAL10* | bifunctional UDP-glucose 4-epimerase/aldose 1-epimerase | -2.76175 | 0.00038 |
| *RME2* | RME2 | -2.77632 | 0.00050 |
| *YFL015C* | hypothetical protein | -2.79819 | 0.02352 |
| *SOR2* | L-iditol 2-dehydrogenase SOR2 | -2.85009 | 0.00053 |
| *SOR1* | L-iditol 2-dehydrogenase SOR1 | -2.85009 | 0.00053 |
| *PRM2* | pheromone-regulated protein PRM2 | -3.0459 | 0.01572 |
| *FAR1* | cyclin-dependent protein serine/threonine kinase inhibiting protein FAR1 | -3.17831 | 0.00010 |
| *RRT5* | Rrt5p | -3.17926 | 0.01810 |
| *YLR227W-B* | gag-pol fusion protein | -3.20696 | 0.00000 |
| *NEJ1* | Nej1p | -3.61084 | 0.00000 |
| *STE18* | Ste18p | -4.18972 | 0.00363 |
| *ICS2* | Ics2p | -4.23557 | 0.00000 |
| *HO* | Hop | -4.32312 | 0.01243 |
| *YHR210C* | aldose 1-epimerase superfamily protein | -4.42378 | 0.04253 |
| *YNL146C-A* | hypothetical protein | -4.49032 | 0.00000 |
| *YLR406C-A* | hypothetical protein | -4.90732 | 0.01449 |
| *MFA2* | mating pheromone a | -5.95918 | 0.00000 |

**Supplementary Figure 1. Aligned Nanopore reads of haploid and diploid cells in the locus of *IME4* gene**. The pink color or the read indicates a sense orientation (from left to right) while the blue color indicates the opposite direction (from right to left). Thanks to the strand information of Nanopore reads, we can see that in the diploid cells, only IME4 is expressed. On the contrary, in the haploid cells, only RME2 is expressed. Without the strand information, it’s not possible to know the parent gene for each read.


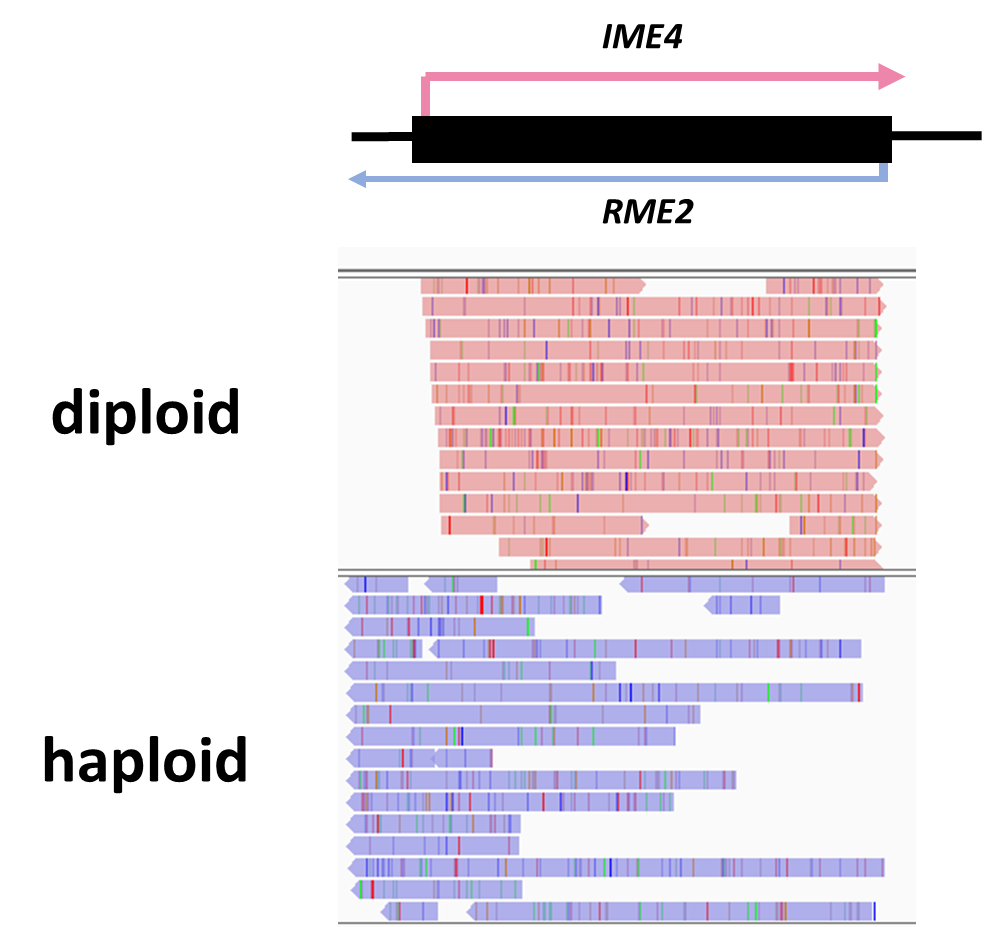


**Supplementary Figure 2. Validation of differential gene expression**. qPCR validated the differential expression for most genes that were found to be differentially expressed by Illumina alone, Nanopore alone or both technologies.

**
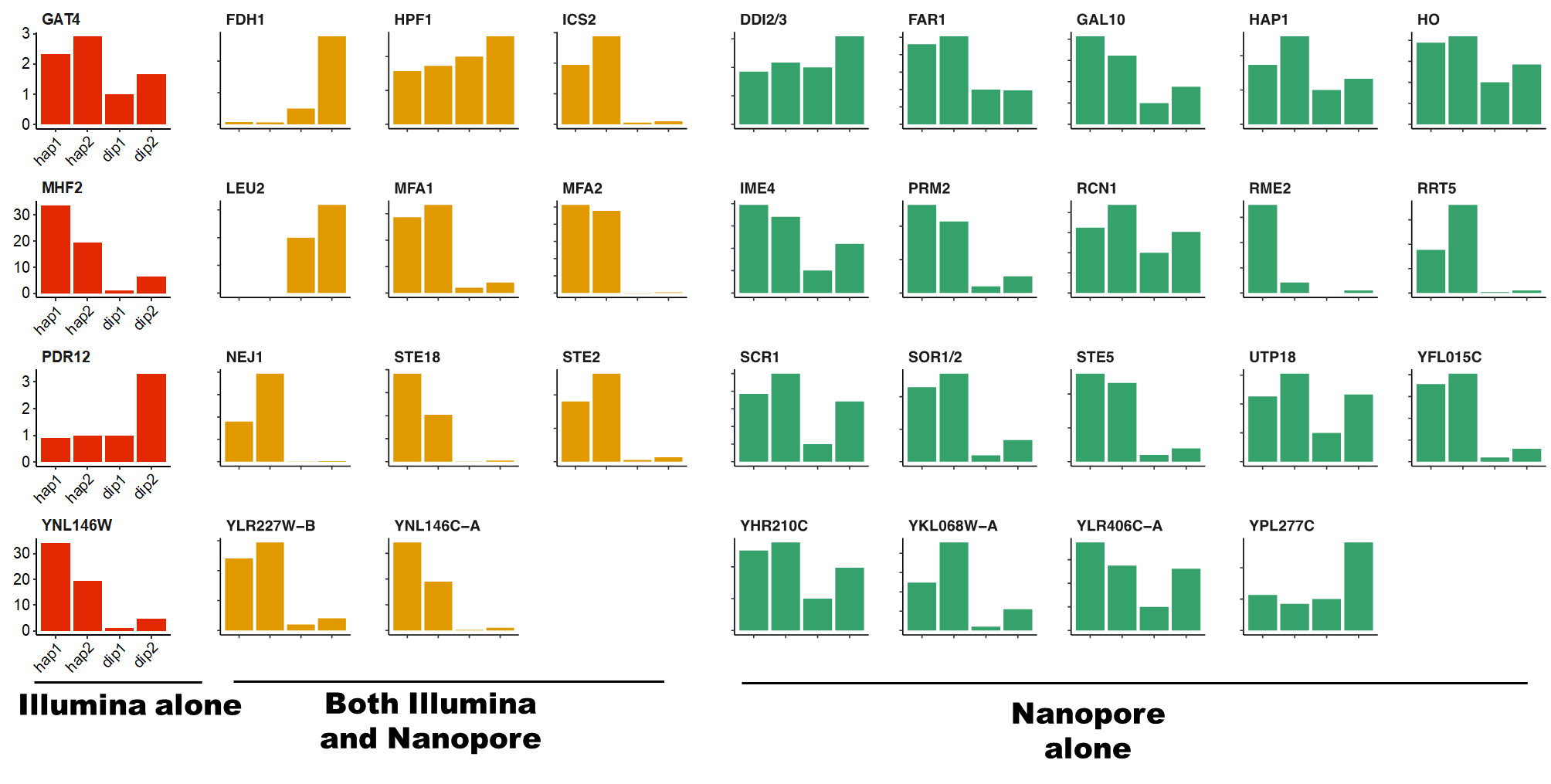
**

**Supplementary figure 3. Coverage of Nanopore and Illumina.** Coverage of Nanopore is more even and uniform through each gene which make it easier to annotate than illumina. The coverage is from diploid Replicate_1, Chr13:133,171-140,526


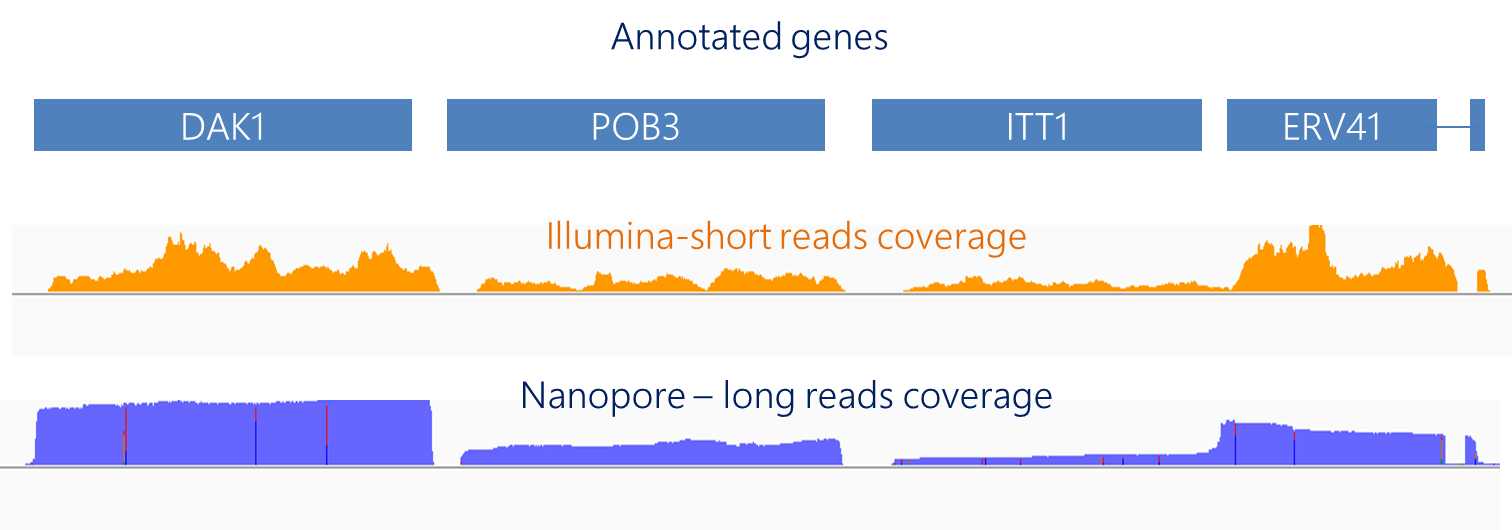


**Supplementary figure 4. An example of high number of duplicates in FLAIR compared to UNAGI to the *ACS1* gene.**

**
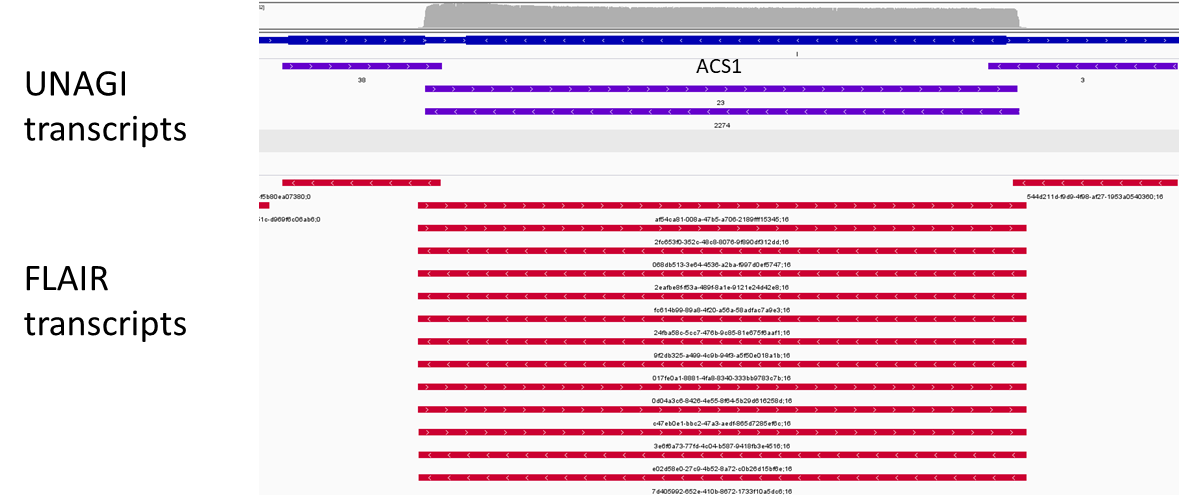
**

**Supplementary figure 5.** An example on lncRNA that overlap with the 5’-untranslated region of *GDH3* leading to a possible interference in transcription

**
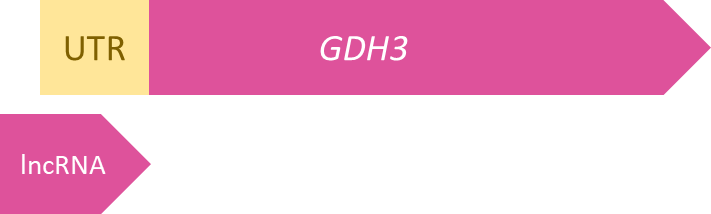
**

**Supplementary figure 6.** COX5B gene. Nanopore reads had artifact insertion after the actual splicing site causing to report a false alternative splicing site(top). the soft-clipped sequence of illumina reads at the splice site is identical to the start of the next exon which conforms this annotated splicing site (down).


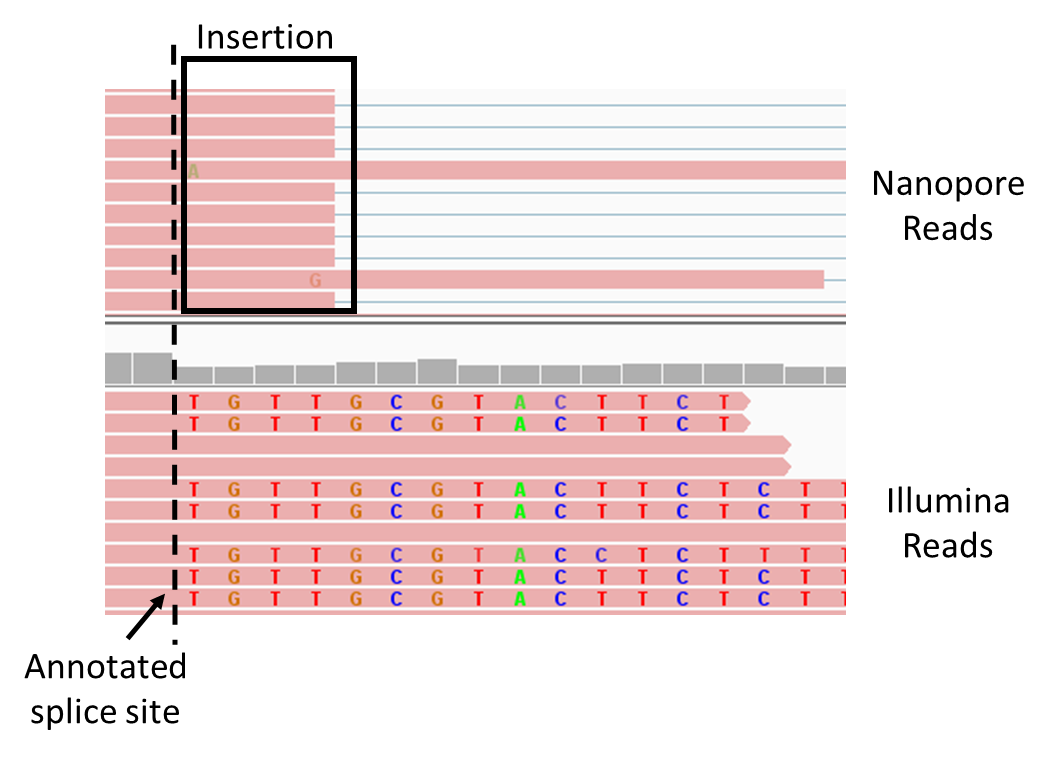


**Supplementary figure 6.** Number of 5’-UTR isoforms per gene. The majority (980 genes) had between 2 to 12 isoforms per gene, 76 genes had 12 to 22 isoforms, 17 genes had 22-32 isoforms, 12 genes had 32-42 isoforms, 3 genes had 42-52 isoforms and one gene had 52 -62 isoforms.


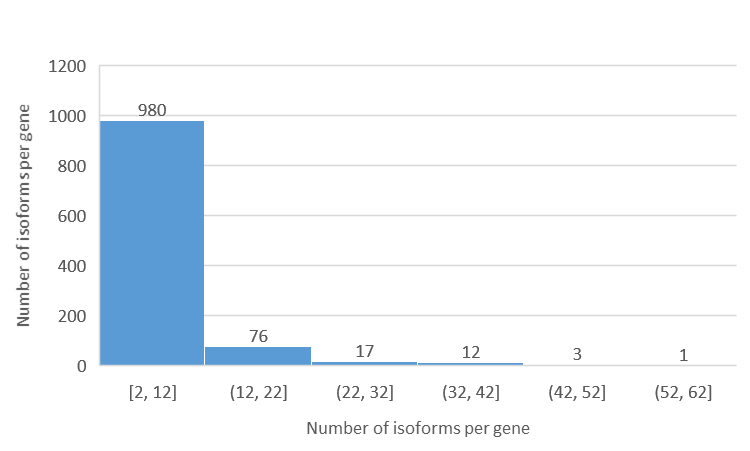


**Supplementary figure 7.** *SNF11* have two isoforms, one of them contains uORF. This may result in higher translation efficiency for the second isoform.


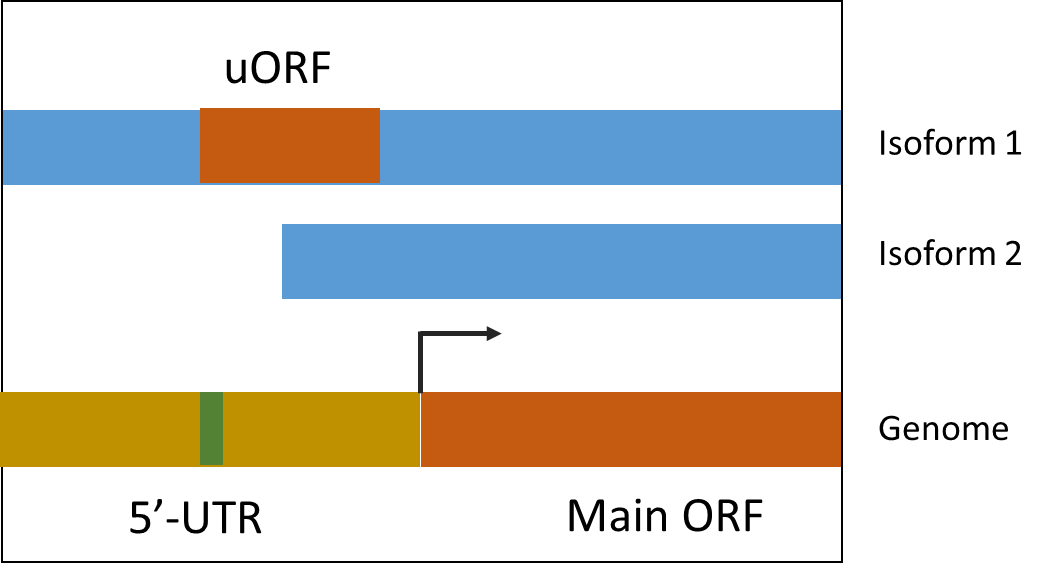

Supplement: Supplementary file 1 — (DOCX 724 kb). [file 10142_2020_732_MOESM1_ESM.docx]
